# Tris(1*H*-imidazole-κ*N* ^3^)(7-oxabicyclo­[2.2.1]heptane-2,3-dicarboxyl­ato-κ^3^ *O* ^2^,*O* ^3^,*O* ^7^)cobalt(II) 3.35-hydrate

**DOI:** 10.1107/S1600536809024039

**Published:** 2009-07-01

**Authors:** Yan-Jun Wang, Rui-Ding Hu, Qiu-Yue Lin, Jian-Ping Cheng

**Affiliations:** a Zhejiang Key Laboratory for Reactive Chemistry on Solid Surfaces, Institute of Physical Chemistry, Zhejiang Normal University, Jinhua, Zhejiang 321004, People’s Republic of China, and, College of Chemistry and Life Science, Zhejiang Normal University, Jinhua 321004, Zhejiang, People’s Republic of China

## Abstract

In the crystal structure of the title compound, [Co(C_8_H_8_O_5_)(C_3_H_4_N_2_)_3_]·3.35H_2_O, the central Co^II^ ion is in a slightly distorted octa­hedral environment, coordinated by the bridg­ing O atom from the bicyclo­[2.2.1]heptane ligand, by two carboxyl­ate O atoms from two different carboxyl­ate groups and by three N atoms from imidazole ligands. Uncoordinated water mol­ecules, some of them disordered, are present in the crystal structure. In the crystal structure, mol­ecules are linked by O—H⋯O, N—H⋯O and O—H⋯N hydrogen-bonding inter­actions.

## Related literature

For several cobalt complexes of norcantharidin, see: Wang *et al.* (1988 ▶) and of imidazole, see: Furenlid *et al.* (1986 ▶); Zhu *et al.* (2003 ▶).
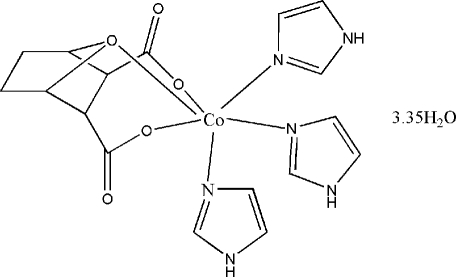


## Experimental

### 

#### Crystal data

[Co(C_8_H_8_O_5_)(C_3_H_4_N_2_)_3_]·3.35H_2_O
*M* *_r_* = 507.67
Triclinic, 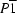

*a* = 8.2666 (2) Å
*b* = 12.6522 (5) Å
*c* = 12.7200 (3) Å
α = 109.912 (2)°
β = 104.394 (1)°
γ = 95.354 (2)°
*V* = 1188.23 (6) Å^3^
*Z* = 2
Mo *K*α radiation
μ = 0.78 mm^−1^
*T* = 296 K
0.41 × 0.36 × 0.29 mm

#### Data collection

Bruker APEXII area-detector diffractometer
Absorption correction: multi-scan (*SADABS*; Sheldrick, 1996 ▶) *T*_min_ = 0.735, *T*_max_ = 0.798
18153 measured reflections
5402 independent reflections
4712 reflections with *I* > 2σ(*I*)
*R*_int_ = 0.024

#### Refinement

*R*[*F* ^2^ > 2σ(*F* ^2^)] = 0.071
*wR*(*F* ^2^) = 0.241
*S* = 1.11
5402 reflections
307 parameters
H-atom parameters constrained
Δρ_max_ = 1.54 e Å^−3^
Δρ_min_ = −0.74 e Å^−3^

### 

Data collection: *APEX2* (Bruker, 2004 ▶); cell refinement: *SAINT* (Bruker, 2004 ▶); data reduction: *SAINT*; program(s) used to solve structure: *SHELXS97* (Sheldrick, 2008 ▶); program(s) used to refine structure: *SHELXL97* (Sheldrick, 2008 ▶); molecular graphics: *SHELXTL* (Sheldrick, 2008 ▶); software used to prepare material for publication: *SHELXL97*.

## Supplementary Material

Crystal structure: contains datablocks I, global. DOI: 10.1107/S1600536809024039/at2795sup1.cif

Structure factors: contains datablocks I. DOI: 10.1107/S1600536809024039/at2795Isup2.hkl

Additional supplementary materials:  crystallographic information; 3D view; checkCIF report

## Figures and Tables

**Table 1:** Hydrogen-bond geometry (Å, °)

*D*—H⋯*A*	*D*—H	H⋯*A*	*D*⋯*A*	*D*—H⋯*A*
O1*W*—H1*WA*⋯O2*W*^i^	0.85	2.33	3.161 (6)	167
O2*W*—H2*WA*⋯N5^ii^	0.85	2.59	3.133 (5)	123
O2*W*—H2*WB*⋯O2*W*^i^	0.85	2.69	3.084 (7)	110
O1*W*—H1*WB*⋯O4	0.85	2.17	2.690 (6)	119
N5—H5*B*⋯O2*W*^ii^	0.86	2.29	3.133 (5)	165

Symmetry codes: (i) 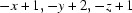; (ii) 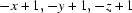.

**Fig. 1.**
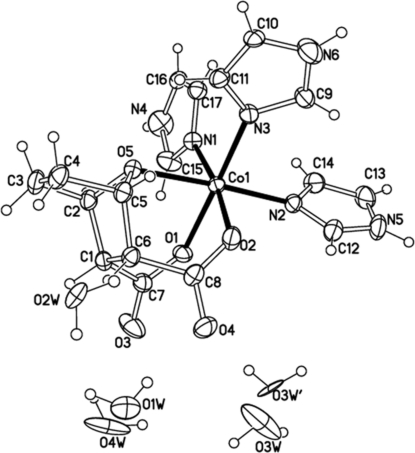
A view of the molecule of (I) showing the atom-labelling scheme with displacement ellipsoids drawn at the 30% probability.

## supplementary crystallographic information

### Comment

7-Oxabicyclo(2,2,1) heptane-2,3-dicarboxylic anhydride (norcantharidin), a traditional Chinese drug, has a great inhibitive effect on common cancer cells. Imidazole is reputed as biocatalyst and biological ligand. Several cobalt complexes of norcantharidin (Wang *et al.*, 1988) and of imidazole (Furenlid *et al.*, 1986; Zhu *et al.*, 2003) have been reported. However, there are no ternary complexes reported about them. So a novel cobalt(II) ternary complex of norcantharidin with imidazole has been synthesized and its single crystals were obtained.

In the title complex, each Co^II^ ion is six-coordinated by one bridge oxygen, two carboxylate oxygen atoms in two different carboxylate groups and three nitrogen atom from imidazoles. O1, O5, N3 and N2 lie in the equatorial plane with the torsion angle 0.06 °. O2 and the nitrogen atom N1 from imidazole are in the axial positions. The bond angle of O2—Co1—N1 is 172.12 (13)°, so it forms a distorted octahedral. Owing to the binding of the bridge oxygen atom with Co, two six-membered rings (Co1/O5/C5/C6/C8/O2 and Co1/O5/C2/C1/C7/O1) are created. In addition, a seven-membered ring (Co1/O2/C8/C6/C1/C7/O1) is formed because of the coordination of carboxylate oxygen atoms O1 and O2, which makes the compound more stable.

In the crystal structure, there are O—H···O and N—H···O and O—H···N hydrogen bonds interactions (Table 1).

### Experimental

A mixture of 0.5 mmol norcantharidin, 0.5 mmol CoCl_2_6H_2_O, 2.5 mmol imidazole and 15 mL distilled water was sealed in a 25 mL Teflon-lined stainless vessel and heated at 443 K for 3 d, then cooled slowly to room temperature. The solution was filtered and after two weeks block orange single crystals were obtained.

### Refinement

The H atoms bonded to C and N atoms were positioned geometrically and refined using a riding model [aromatic C—H = 0.93 Å, aliphatic C—H = 0.97 (2) Å and N—H = 0.86 Å, *U*_iso_(H) = 1.2*U*_eq_(C)]. The H atoms bonded to O atoms were located in a difference Fourier maps and refined with O—H distance restraints of 0.85 (2) and *U*_iso_(H) = 1.5*U*_eq_(O).

### Crystal data

**Table:**

[Co(C_8_H_8_O_5_)(C_3_H_4_N_2_)_3_]·3.35H_2_O	*Z* = 2
*M**_r_* = 507.67	*F*(000) = 529
Triclinic, *P*1	*D*_x_ = 1.419 Mg m^−^^3^
Hall symbol: -P 1	Mo *K*α radiation, λ = 0.71073 Å
*a* = 8.2666 (2) Å	Cell parameters from 8576 reflections
*b* = 12.6522 (5) Å	θ = 1.8–27.7°
*c* = 12.7200 (3) Å	µ = 0.78 mm^−^^1^
α = 109.912 (2)°	*T* = 296 K
β = 104.394 (1)°	Block, orange
γ = 95.354 (2)°	0.41 × 0.36 × 0.29 mm
*V* = 1188.23 (6) Å^3^	

### Data collection

**Table:**

Bruker APEXII area-detector diffractometer	5402 independent reflections
Radiation source: fine-focus sealed tube	4712 reflections with *I* > 2σ(*I*)
graphite	*R*_int_ = 0.024
ω scans	θ_max_ = 27.7°, θ_min_ = 1.8°
Absorption correction: multi-scan (*SADABS*; Sheldrick, 1996)	*h* = −10→10
*T*_min_ = 0.735, *T*_max_ = 0.798	*k* = −15→16
18153 measured reflections	*l* = −16→16

### Refinement

**Table:**

Refinement on *F*^2^	Primary atom site location: structure-invariant direct methods
Least-squares matrix: full	Secondary atom site location: difference Fourier map
*R*[*F*^2^ > 2σ(*F*^2^)] = 0.071	Hydrogen site location: inferred from neighbouring sites
*wR*(*F*^2^) = 0.241	H-atom parameters constrained
*S* = 1.11	*w* = 1/[σ^2^(*F*_o_^2^) + (0.1424*P*)^2^ + 2.537*P*] where *P* = (*F*_o_^2^ + 2*F*_c_^2^)/3
5402 reflections	(Δ/σ)_max_ = 0.001
307 parameters	Δρ_max_ = 1.54 e Å^−^^3^
0 restraints	Δρ_min_ = −0.74 e Å^−^^3^

### Special details

**Table:**

Geometry. All e.s.d.'s (except the e.s.d. in the dihedral angle between two l.s. planes) are estimated using the full covariance matrix. The cell e.s.d.'s are taken into account individually in the estimation of e.s.d.'s in distances, angles and torsion angles; correlations between e.s.d.'s in cell parameters are only used when they are defined by crystal symmetry. An approximate (isotropic) treatment of cell e.s.d.'s is used for estimating e.s.d.'s involving l.s. planes.
Refinement. Refinement of *F*^2^ against ALL reflections. The weighted *R*-factor *wR* and goodness of fit *S* are based on *F*^2^, conventional *R*-factors *R* are based on *F*, with *F* set to zero for negative *F*^2^. The threshold expression of *F*^2^ > σ(*F*^2^) is used only for calculating *R*-factors(gt) *etc*. and is not relevant to the choice of reflections for refinement. *R*-factors based on *F*^2^ are statistically about twice as large as those based on *F*, and *R*- factors based on ALL data will be even larger.

### Fractional atomic coordinates and isotropic or equivalent isotropic displacement parameters (Å^2^)

**Table:**

	*x*	*y*	*z*	*U*_iso_*/*U*_eq_	Occ. (<1)
Co1	0.63081 (6)	0.68205 (4)	0.83619 (4)	0.0284 (2)	
N1	0.7319 (4)	0.7409 (3)	1.0204 (3)	0.0341 (7)	
N2	0.6963 (5)	0.5190 (3)	0.8077 (3)	0.0346 (7)	
N3	0.3830 (4)	0.6199 (3)	0.8333 (3)	0.0349 (7)	
N4	0.9131 (7)	0.8220 (5)	1.2013 (5)	0.0693 (14)	
H4C	1.0039	0.8564	1.2590	0.083*	
N5	0.6939 (6)	0.3370 (3)	0.7193 (5)	0.0574 (12)	
H5B	0.6793	0.2715	0.6638	0.069*	
N6	0.1391 (6)	0.5038 (5)	0.7969 (5)	0.0643 (13)	
H6B	0.0643	0.4437	0.7798	0.077*	
O1	0.8683 (4)	0.7508 (3)	0.8304 (3)	0.0391 (7)	
O1W	0.7281 (7)	0.8341 (4)	0.4258 (4)	0.0742 (13)	
H1WA	0.7302	0.9031	0.4678	0.089*	
H1WB	0.6385	0.7925	0.4222	0.089*	
O2W	0.3268 (4)	0.9162 (3)	0.4450 (2)	0.0376 (7)	
H2WA	0.2966	0.8498	0.4443	0.045*	
H2WB	0.4062	0.9147	0.4132	0.045*	
O2	0.5497 (5)	0.6445 (3)	0.6545 (3)	0.0451 (8)	
O3	1.0444 (5)	0.8533 (4)	0.7804 (5)	0.0725 (14)	
O3W	1.091 (2)	0.5698 (11)	0.5348 (6)	0.191 (9)	0.62
H3WA	1.1554	0.5213	0.5352	0.229*	0.62
H3WB	1.1423	0.6254	0.5251	0.229*	0.62
O3W'	0.9060 (13)	0.5536 (8)	0.5087 (6)	0.044 (2)	0.38
H4WA	0.8377	0.5995	0.5196	0.052*	0.38
H4WB	0.8626	0.4913	0.5114	0.052*	0.38
O4W	1.0657 (12)	0.8869 (16)	0.5732 (9)	0.091 (6)	0.35
H5WA	1.0732	0.9208	0.6450	0.110*	0.35
H5WB	1.0588	0.8156	0.5590	0.110*	0.35
O4	0.6568 (6)	0.6890 (3)	0.5285 (3)	0.0546 (9)	
O5	0.5636 (4)	0.8523 (2)	0.8560 (2)	0.0317 (6)	
C1	0.7756 (5)	0.9070 (3)	0.7836 (4)	0.0356 (8)	
H1A	0.8265	0.9744	0.7730	0.043*	
C2	0.7068 (5)	0.9451 (3)	0.8892 (4)	0.0362 (9)	
H2A	0.7914	0.9584	0.9640	0.043*	
C3	0.6172 (7)	1.0455 (4)	0.8901 (5)	0.0495 (12)	
H3A	0.6853	1.1039	0.8772	0.059*	
H3B	0.5916	1.0798	0.9634	0.059*	
C4	0.4520 (7)	0.9865 (4)	0.7860 (5)	0.0472 (11)	
H4A	0.3510	0.9953	0.8114	0.057*	
H4B	0.4464	1.0163	0.7246	0.057*	
C5	0.4739 (5)	0.8612 (3)	0.7458 (4)	0.0343 (8)	
H5A	0.3661	0.8060	0.7038	0.041*	
C6	0.6067 (5)	0.8427 (3)	0.6801 (4)	0.0342 (8)	
H6A	0.5887	0.8810	0.6241	0.041*	
C7	0.9075 (5)	0.8317 (4)	0.7984 (4)	0.0385 (9)	
C8	0.6040 (6)	0.7161 (4)	0.6169 (4)	0.0371 (9)	
C9	0.3052 (6)	0.5083 (4)	0.8036 (5)	0.0453 (11)	
H9A	0.3589	0.4457	0.7901	0.054*	
C10	0.1132 (5)	0.6143 (4)	0.8230 (4)	0.0339 (8)	
H10A	0.0110	0.6385	0.8261	0.041*	
C11	0.2615 (5)	0.6804 (4)	0.8431 (4)	0.0380 (9)	
H11A	0.2776	0.7591	0.8617	0.046*	
C12	0.6597 (6)	0.4343 (4)	0.7060 (5)	0.0455 (10)	
H12A	0.6159	0.4406	0.6341	0.055*	
C13	0.7553 (8)	0.3606 (5)	0.8352 (6)	0.0592 (15)	
H13A	0.7900	0.3096	0.8703	0.071*	
C14	0.7564 (7)	0.4729 (4)	0.8902 (5)	0.0479 (11)	
H14A	0.7921	0.5128	0.9709	0.057*	
C15	0.8950 (6)	0.7955 (5)	1.0852 (4)	0.0519 (12)	
H15A	0.9810	0.8122	1.0548	0.062*	
C16	0.7597 (6)	0.7835 (4)	1.2069 (3)	0.0349 (8)	
H16A	0.7321	0.7890	1.2749	0.042*	
C17	0.6539 (6)	0.7360 (4)	1.0978 (4)	0.0404 (9)	
H17A	0.5397	0.7035	1.0787	0.048*	

### Atomic displacement parameters (Å^2^)

**Table:**

	*U*^11^	*U*^22^	*U*^33^	*U*^12^	*U*^13^	*U*^23^
Co1	0.0262 (3)	0.0286 (3)	0.0308 (3)	0.0069 (2)	0.0089 (2)	0.0113 (2)
N1	0.0325 (17)	0.0372 (17)	0.0323 (17)	0.0075 (14)	0.0079 (14)	0.0141 (14)
N2	0.0330 (17)	0.0310 (16)	0.0418 (18)	0.0094 (13)	0.0126 (14)	0.0142 (14)
N3	0.0270 (16)	0.0350 (17)	0.0418 (18)	0.0065 (13)	0.0086 (14)	0.0148 (15)
N4	0.070 (3)	0.075 (3)	0.048 (3)	0.001 (3)	0.001 (2)	0.019 (2)
N5	0.059 (3)	0.0284 (19)	0.078 (3)	0.0115 (18)	0.023 (2)	0.009 (2)
N6	0.046 (2)	0.061 (3)	0.086 (4)	0.000 (2)	0.021 (2)	0.030 (3)
O1	0.0350 (15)	0.0425 (16)	0.0522 (18)	0.0137 (13)	0.0216 (14)	0.0251 (14)
O1W	0.099 (4)	0.078 (3)	0.060 (3)	0.020 (3)	0.043 (3)	0.029 (2)
O2W	0.0422 (16)	0.0350 (14)	0.0265 (13)	0.0221 (13)	0.0029 (12)	0.0021 (11)
O2	0.066 (2)	0.0317 (15)	0.0354 (16)	0.0101 (14)	0.0132 (15)	0.0111 (12)
O3	0.040 (2)	0.081 (3)	0.134 (4)	0.0207 (19)	0.043 (2)	0.071 (3)
O3W	0.271 (17)	0.180 (11)	0.027 (3)	−0.178 (12)	−0.009 (6)	0.020 (5)
O3W'	0.069 (6)	0.063 (5)	0.022 (3)	0.060 (5)	0.029 (4)	0.021 (3)
O4W	0.027 (5)	0.205 (17)	0.031 (5)	−0.031 (7)	−0.004 (4)	0.054 (8)
O4	0.080 (3)	0.050 (2)	0.0413 (18)	0.0216 (18)	0.0306 (18)	0.0152 (15)
O5	0.0320 (14)	0.0287 (13)	0.0330 (14)	0.0067 (11)	0.0086 (11)	0.0103 (11)
C1	0.037 (2)	0.0283 (18)	0.044 (2)	0.0063 (15)	0.0145 (17)	0.0140 (16)
C2	0.035 (2)	0.0288 (18)	0.038 (2)	0.0029 (15)	0.0080 (17)	0.0069 (16)
C3	0.057 (3)	0.027 (2)	0.059 (3)	0.0092 (19)	0.019 (2)	0.0073 (19)
C4	0.052 (3)	0.040 (2)	0.055 (3)	0.024 (2)	0.018 (2)	0.019 (2)
C5	0.034 (2)	0.0325 (19)	0.035 (2)	0.0105 (15)	0.0073 (16)	0.0122 (16)
C6	0.041 (2)	0.0323 (19)	0.0319 (19)	0.0131 (16)	0.0100 (16)	0.0139 (16)
C7	0.032 (2)	0.038 (2)	0.046 (2)	0.0063 (16)	0.0137 (18)	0.0167 (18)
C8	0.044 (2)	0.037 (2)	0.0276 (18)	0.0141 (17)	0.0061 (16)	0.0104 (16)
C9	0.038 (2)	0.038 (2)	0.067 (3)	0.0092 (18)	0.022 (2)	0.024 (2)
C10	0.0213 (16)	0.040 (2)	0.040 (2)	0.0085 (15)	0.0111 (15)	0.0134 (17)
C11	0.033 (2)	0.039 (2)	0.041 (2)	0.0093 (17)	0.0128 (17)	0.0109 (17)
C12	0.046 (3)	0.037 (2)	0.049 (3)	0.0099 (19)	0.014 (2)	0.0108 (19)
C13	0.069 (4)	0.046 (3)	0.086 (4)	0.026 (3)	0.036 (3)	0.041 (3)
C14	0.054 (3)	0.049 (3)	0.055 (3)	0.022 (2)	0.022 (2)	0.030 (2)
C15	0.036 (2)	0.071 (3)	0.041 (2)	−0.004 (2)	0.0040 (19)	0.021 (2)
C16	0.047 (2)	0.035 (2)	0.0255 (17)	0.0134 (17)	0.0125 (16)	0.0135 (15)
C17	0.043 (2)	0.041 (2)	0.041 (2)	0.0109 (18)	0.0162 (19)	0.0179 (19)

### Geometric parameters (Å, °)

**Table:**

Co1—O1	2.101 (3)	O4W—H5WB	0.8502
Co1—O2	2.107 (3)	O4—C8	1.258 (5)
Co1—N3	2.112 (3)	O5—C2	1.453 (5)
Co1—N1	2.113 (3)	O5—C5	1.461 (5)
Co1—N2	2.116 (3)	C1—C7	1.531 (6)
Co1—O5	2.222 (3)	C1—C2	1.534 (6)
N1—C17	1.319 (6)	C1—C6	1.578 (6)
N1—C15	1.364 (6)	C1—H1A	0.9800
N2—C12	1.310 (6)	C2—C3	1.528 (6)
N2—C14	1.378 (6)	C2—H2A	0.9800
N3—C11	1.323 (5)	C3—C4	1.553 (8)
N3—C9	1.377 (6)	C3—H3A	0.9700
N4—C16	1.343 (7)	C3—H3B	0.9700
N4—C15	1.363 (7)	C4—C5	1.534 (6)
N4—H4C	0.8600	C4—H4A	0.9700
N5—C12	1.344 (7)	C4—H4B	0.9700
N5—C13	1.350 (8)	C5—C6	1.526 (6)
N5—H5B	0.8600	C5—H5A	0.9800
N6—C9	1.349 (7)	C6—C8	1.523 (6)
N6—C10	1.374 (7)	C6—H6A	0.9800
N6—H6B	0.8600	C9—H9A	0.9300
O1—C7	1.268 (5)	C10—C11	1.333 (6)
O1W—H1WA	0.8501	C10—H10A	0.9300
O1W—H1WB	0.8499	C11—H11A	0.9300
O2W—H2WA	0.8500	C12—H12A	0.9300
O2W—H2WB	0.8503	C13—C14	1.351 (7)
O2—C8	1.257 (6)	C13—H13A	0.9300
O3—C7	1.234 (6)	C14—H14A	0.9300
O3W—H3WA	0.8500	C15—H15A	0.9300
O3W—H3WB	0.8498	C16—C17	1.336 (6)
O3W'—H4WA	0.8499	C16—H16A	0.9300
O3W'—H4WB	0.8502	C17—H17A	0.9300
O4W—H5WA	0.8500		
			
O1—Co1—O2	85.20 (14)	C2—C3—H3A	111.4
O1—Co1—N3	175.35 (12)	C4—C3—H3A	111.4
O2—Co1—N3	91.49 (15)	C2—C3—H3B	111.4
O1—Co1—N1	88.60 (14)	C4—C3—H3B	111.4
O2—Co1—N1	172.12 (13)	H3A—C3—H3B	109.2
N3—Co1—N1	94.41 (14)	C5—C4—C3	101.3 (4)
O1—Co1—N2	91.64 (13)	C5—C4—H4A	111.5
O2—Co1—N2	91.04 (13)	C3—C4—H4A	111.5
N3—Co1—N2	91.68 (14)	C5—C4—H4B	111.5
N1—Co1—N2	93.99 (14)	C3—C4—H4B	111.5
O1—Co1—O5	86.79 (11)	H4A—C4—H4B	109.3
O2—Co1—O5	85.90 (11)	O5—C5—C6	101.7 (3)
N3—Co1—O5	89.72 (12)	O5—C5—C4	102.2 (3)
N1—Co1—O5	88.91 (12)	C6—C5—C4	111.2 (4)
N2—Co1—O5	176.67 (12)	O5—C5—H5A	113.5
C17—N1—C15	104.9 (4)	C6—C5—H5A	113.5
C17—N1—Co1	128.7 (3)	C4—C5—H5A	113.5
C15—N1—Co1	126.4 (3)	C8—C6—C5	112.3 (4)
C12—N2—C14	105.6 (4)	C8—C6—C1	113.4 (3)
C12—N2—Co1	125.7 (3)	C5—C6—C1	101.0 (3)
C14—N2—Co1	127.8 (3)	C8—C6—H6A	110.0
C11—N3—C9	105.4 (4)	C5—C6—H6A	110.0
C11—N3—Co1	125.4 (3)	C1—C6—H6A	110.0
C9—N3—Co1	128.4 (3)	O3—C7—O1	124.0 (4)
C16—N4—C15	105.8 (5)	O3—C7—C1	119.2 (4)
C16—N4—H4C	127.1	O1—C7—C1	116.9 (4)
C15—N4—H4C	127.1	O2—C8—O4	123.3 (4)
C12—N5—C13	107.7 (4)	O2—C8—C6	119.2 (4)
C12—N5—H5B	126.2	O4—C8—C6	117.4 (4)
C13—N5—H5B	126.2	N6—C9—N3	109.5 (4)
C9—N6—C10	106.3 (4)	N6—C9—H9A	125.2
C9—N6—H6B	126.9	N3—C9—H9A	125.2
C10—N6—H6B	126.9	C11—C10—N6	107.3 (4)
C7—O1—Co1	129.5 (3)	C11—C10—H10A	126.3
H1WA—O1W—H1WB	108.4	N6—C10—H10A	126.3
H2WA—O2W—H2WB	108.8	N3—C11—C10	111.4 (4)
C8—O2—Co1	118.5 (3)	N3—C11—H11A	124.3
H3WA—O3W—H3WB	108.3	C10—C11—H11A	124.3
H4WA—O3W'—H4WB	108.1	N2—C12—N5	111.1 (5)
H5WA—O4W—H5WB	108.2	N2—C12—H12A	124.5
C2—O5—C5	96.4 (3)	N5—C12—H12A	124.5
C2—O5—Co1	115.2 (2)	N5—C13—C14	106.5 (5)
C5—O5—Co1	114.1 (2)	N5—C13—H13A	126.8
C7—C1—C2	111.4 (4)	C14—C13—H13A	126.8
C7—C1—C6	114.2 (3)	C13—C14—N2	109.2 (5)
C2—C1—C6	101.4 (3)	C13—C14—H14A	125.4
C7—C1—H1A	109.9	N2—C14—H14A	125.4
C2—C1—H1A	109.9	N4—C15—N1	109.9 (5)
C6—C1—H1A	109.9	N4—C15—H15A	125.1
O5—C2—C3	101.6 (3)	N1—C15—H15A	125.1
O5—C2—C1	102.2 (3)	C17—C16—N4	108.0 (4)
C3—C2—C1	110.4 (4)	C17—C16—H16A	126.0
O5—C2—H2A	113.8	N4—C16—H16A	126.0
C3—C2—H2A	113.8	N1—C17—C16	111.4 (4)
C1—C2—H2A	113.8	N1—C17—H17A	124.3
C2—C3—C4	102.0 (3)	C16—C17—H17A	124.3
			
O1—Co1—N1—C17	176.2 (4)	C1—C2—C3—C4	71.7 (5)
N3—Co1—N1—C17	−0.2 (4)	C2—C3—C4—C5	1.5 (5)
N2—Co1—N1—C17	−92.2 (4)	C2—O5—C5—C6	58.6 (3)
O5—Co1—N1—C17	89.4 (4)	Co1—O5—C5—C6	−62.7 (3)
O1—Co1—N1—C15	−2.2 (4)	C2—O5—C5—C4	−56.5 (4)
N3—Co1—N1—C15	−178.6 (4)	Co1—O5—C5—C4	−177.8 (3)
N2—Co1—N1—C15	89.4 (4)	C3—C4—C5—O5	33.4 (5)
O5—Co1—N1—C15	−89.0 (4)	C3—C4—C5—C6	−74.5 (5)
O1—Co1—N2—C12	−94.7 (4)	O5—C5—C6—C8	84.1 (4)
O2—Co1—N2—C12	−9.4 (4)	C4—C5—C6—C8	−167.7 (4)
N3—Co1—N2—C12	82.1 (4)	O5—C5—C6—C1	−37.0 (4)
N1—Co1—N2—C12	176.6 (4)	C4—C5—C6—C1	71.2 (4)
O1—Co1—N2—C14	98.0 (4)	C7—C1—C6—C8	2.1 (5)
O2—Co1—N2—C14	−176.7 (4)	C2—C1—C6—C8	−117.8 (4)
N3—Co1—N2—C14	−85.2 (4)	C7—C1—C6—C5	122.4 (4)
N1—Co1—N2—C14	9.3 (4)	C2—C1—C6—C5	2.4 (4)
O2—Co1—N3—C11	−86.7 (4)	Co1—O1—C7—O3	−167.8 (4)
N1—Co1—N3—C11	88.1 (4)	Co1—O1—C7—C1	13.2 (6)
N2—Co1—N3—C11	−177.7 (4)	C2—C1—C7—O3	−131.4 (5)
O5—Co1—N3—C11	−0.8 (4)	C6—C1—C7—O3	114.4 (5)
O2—Co1—N3—C9	82.4 (4)	C2—C1—C7—O1	47.7 (5)
N1—Co1—N3—C9	−102.9 (4)	C6—C1—C7—O1	−66.4 (5)
N2—Co1—N3—C9	−8.7 (4)	Co1—O2—C8—O4	135.3 (4)
O5—Co1—N3—C9	168.3 (4)	Co1—O2—C8—C6	−44.8 (5)
O2—Co1—O1—C7	59.9 (4)	C5—C6—C8—O2	−25.7 (5)
N1—Co1—O1—C7	−115.3 (4)	C1—C6—C8—O2	88.0 (5)
N2—Co1—O1—C7	150.8 (4)	C5—C6—C8—O4	154.1 (4)
O5—Co1—O1—C7	−26.3 (4)	C1—C6—C8—O4	−92.2 (5)
O1—Co1—O2—C8	−38.7 (3)	C10—N6—C9—N3	0.0 (6)
N3—Co1—O2—C8	138.0 (4)	C11—N3—C9—N6	−0.5 (6)
N2—Co1—O2—C8	−130.3 (3)	Co1—N3—C9—N6	−171.2 (4)
O5—Co1—O2—C8	48.4 (3)	C9—N6—C10—C11	0.5 (6)
O1—Co1—O5—C2	−16.1 (3)	C9—N3—C11—C10	0.8 (5)
O2—Co1—O5—C2	−101.6 (3)	Co1—N3—C11—C10	171.9 (3)
N3—Co1—O5—C2	166.9 (3)	N6—C10—C11—N3	−0.9 (6)
N1—Co1—O5—C2	72.5 (3)	C14—N2—C12—N5	−0.2 (6)
O1—Co1—O5—C5	94.0 (3)	Co1—N2—C12—N5	−169.8 (3)
O2—Co1—O5—C5	8.6 (3)	C13—N5—C12—N2	0.0 (6)
N3—Co1—O5—C5	−82.9 (3)	C12—N5—C13—C14	0.2 (6)
N1—Co1—O5—C5	−177.3 (3)	N5—C13—C14—N2	−0.3 (6)
C5—O5—C2—C3	57.3 (4)	C12—N2—C14—C13	0.4 (6)
Co1—O5—C2—C3	177.8 (3)	Co1—N2—C14—C13	169.7 (4)
C5—O5—C2—C1	−56.8 (3)	C16—N4—C15—N1	−0.2 (7)
Co1—O5—C2—C1	63.7 (3)	C17—N1—C15—N4	0.4 (6)
C7—C1—C2—O5	−88.8 (4)	Co1—N1—C15—N4	179.2 (4)
C6—C1—C2—O5	33.1 (4)	C15—N4—C16—C17	−0.2 (6)
C7—C1—C2—C3	163.7 (4)	C15—N1—C17—C16	−0.6 (5)
C6—C1—C2—C3	−74.4 (4)	Co1—N1—C17—C16	−179.2 (3)
O5—C2—C3—C4	−36.2 (5)	N4—C16—C17—N1	0.5 (6)

### Hydrogen-bond geometry (Å, °)

**Table:**

*D*—H···*A*	*D*—H	H···*A*	*D*···*A*	*D*—H···*A*
O1W—H1WA···O2W^i^	0.85	2.33	3.161 (6)	167
O2W—H2WA···N5^ii^	0.85	2.59	3.133 (5)	123
O2W—H2WB···O2W^i^	0.85	2.69	3.084 (7)	110
O1W—H1WB···O4	0.85	2.17	2.690 (6)	119
N5—H5B···O2W^ii^	0.86	2.29	3.133 (5)	165

Symmetry codes: (i) −*x*+1, −*y*+2, −*z*+1; (ii) −*x*+1, −*y*+1, −*z*+1.
